# *γCOP* Is Required for Apical Protein Secretion and Epithelial Morphogenesis in *Drosophila melanogaster*

**DOI:** 10.1371/journal.pone.0003241

**Published:** 2008-09-19

**Authors:** Nicole C. Grieder, Emmanuel Caussinus, David S. Parker, Kenneth Cadigan, Markus Affolter, Stefan Luschnig

**Affiliations:** 1 Abteilung Zellbiologie, Biozentrum der Universität Basel, Basel, Switzerland; 2 Department of Molecular, Cellular and Developmental Biology, University of Michigan, Ann Arbor, Michigan, United States of America; 3 Developmental Biology, Institute of Zoology, University of Zürich, Zürich, Switzerland; University of Geveva, Switzerland

## Abstract

**Background:**

There is increasing evidence that tissue-specific modifications of basic cellular functions play an important role in development and disease. To identify the functions of COPI coatomer-mediated membrane trafficking in *Drosophila* development, we were aiming to create loss-of-function mutations in the *γCOP* gene, which encodes a subunit of the COPI coatomer complex.

**Principal Findings:**

We found that *γCOP* is essential for the viability of the *Drosophila* embryo. In the absence of zygotic *γCOP* activity, embryos die late in embryogenesis and display pronounced defects in morphogenesis of the embryonic epidermis and of tracheal tubes. The coordinated cell rearrangements and cell shape changes during tracheal tube morphogenesis critically depend on apical secretion of certain proteins. Investigation of tracheal morphogenesis in *γCOP* loss-of-function mutants revealed that several key proteins required for tracheal morphogenesis are not properly secreted into the apical lumen. As a consequence, *γCOP* mutants show defects in cell rearrangements during branch elongation, in tube dilation, as well as in tube fusion. We present genetic evidence that a specific subset of the tracheal defects in *γCOP* mutants is due to the reduced secretion of the Zona Pellucida protein Piopio. Thus, we identified a critical target protein of COPI-dependent secretion in epithelial tube morphogenesis.

**Conclusions/Significance:**

These studies highlight the role of COPI coatomer-mediated vesicle trafficking in both general and tissue-specific secretion in a multicellular organism. Although COPI coatomer is generally required for protein secretion, we show that the phenotypic effect of *γCOP* mutations is surprisingly specific. Importantly, we attribute a distinct aspect of the *γCOP* phenotype to the effect on a specific key target protein.

## Introduction

Many organs are composed of sheets or tubes of epithelial cells. Epithelia create a diffusion barrier and at the same time mediate selective transport of substances within organs. These functions depend on proper apical-basal polarization of epithelia. In the case of tubular organs, such as the lungs or kidneys, the apical epithelial surface faces the tube lumen, and the basal side forms the outside of the tubes [1]. It is of key importance for organogenesis and for proper function of the mature organ that secreted proteins and membrane material are transported to their correct (apical or basal) destinations at the right time. Thus, the spatiotemporal control of secretion plays a crucial role in organ development and physiology. Yet, these processes have been studied mainly using *in vitro* tissue culture models, and functional studies *in vivo* have thus far been rare [2]–[4].

The *Drosophila* tracheal system, a network of gas-filled epithelial tubes, has emerged as a powerful model to study the cellular and molecular basis of tubular organ development *in vivo* [5]–[7]. Tracheal tubes originate from segmentally repeated clusters of epidermal cells that invaginate and subsequently branch out to form a network of interconnected tubes that supply oxygen to target tissues. Importantly, tracheal morphogenesis occurs in the absence of cell division and relies entirely on coordinated changes in cell shape and cell rearrangements. Several steps of this morphogenetic program were recently shown to critically depend on apical protein secretion. First, secretion of two Zona Pellucida (ZP)-domain proteins, Piopio (Pio) and Dumpy (Dp), into the luminal space was shown to be critical for proper cell rearrangement during branch elongation [8]. In the absence of Pio or Dp, branches disconnect from each other and form cyst-like structures. Second, when adjacent tracheal metameres fuse to give rise to interconnected tubes, pairs of specialized cells at the tips of neighboring branches contact each other and form new apical lumens that grow towards each other and eventually fuse, resulting in a continuous lumen [9]–[10]. The formation of the fusion cell lumen was shown to depend on targeted exocytosis and local plasma membrane remodeling [11] mediated by the Arf-like 3 small GTPase [12]–[13]. It was suggested that the exocyst complex controls the assembly of the specialized fusion cell lumen. Third, upon completion of tracheal tube fusion in the embryo, the initially narrow lumen expands to its final size to allow for efficient gas transport in the larva. Tube expansion occurs rapidly within a few hours. During this process, the apical (luminal) surface of tracheal cells grows selectively, while the basal surface shows little change, thus resulting in an expansion of luminal diameter and a flattening of tracheal cells [14]. This expansion phase is temporally coupled with a peak in secretory activity of tracheal cells [2]. Just before and during expansion, large amounts of proteins are secreted into the lumen, where they form an apical extracellular matrix (aECM). This matrix, which contains the polysaccharide chitin in addition to secreted proteins, plays important roles in controlling the shape and size of tracheal tubes. The aECM components Serpentine (Serp) and Vermiform (Verm) are predicted chitin-binding proteins required for limiting tracheal tube elongation [15]–[17]. In contrast, chitin forms a luminal scaffold that appears to be required for uniform expansion of tube diameter [18]–[20]. Components of the COPII vesicle traffic machinery (the GTPase Sar1 and the COPII coat proteins Sec13 and Sec23), which exports proteins from the Endoplasmic Reticulum (ER) to the Golgi complex, were shown to be required for apical secretion of certain aECM components [2]. However, the precise function of the secretory apparatus in tube expansion, as well as the identity of the secreted factors required for proper tube morphogenesis, are not yet known.

The COPI coatomer complex is involved in membrane traffic of small vesicles. Coatomer is trafficking primarily from the early Golgi to the ER and is found on vesicles derived from Golgi cisternae [21]–[22]. Other intracellular routes have also been proposed [21], [23]–[24]. For example, COPI coated vesicles have been proposed to play a role in peroxisome biogenesis and peroxisome to ER transport [24]. In addition, coatomer is directed to the nuclear membrane by the nuclear pore protein Nup153 at mitosis [25].

COPI coatomer was characterized as a large heptameric complex, conserved from yeast to mammals [26]. It contains the α, β, β', γ, δ, ε and ζCOP subunits. β, γ, δ and ζCOP share a distant homology with AP clathrin adaptor subunits [27]. αCOP and β'COP are WD40 proteins [28]. Cytosolic coatomer is recruited to membranes *en bloc* upon stimulation by the membrane-associated, GTP-bound form of the small myristoylated G protein ARF (adenosine-diphosphate-ribosylation factor). Coat disassembly is triggered by an ARF-GTPase activating enzyme (GAP) [22], [26]. In addition to ARF, the p23 and the p24 type I membrane proteins play a role in coat formation and in cargo selection [21]. Coatomer is recruited to membranes through interaction of ARF with the β− and the γCOP subunit and also through interaction of the γCOP subunit with p23 or p24, which are also involved in ARF recruitment [21]. COPI coatomer-coated vesicles contain cargo indicative of both forward and retrograde transport. Thus, there must be mechanisms determining the content and the various destinations of different COPI coated vesicles. Coating vesicles with distinct combinations of different isotypic coatomer subunits may assist sorting to various destinations and may also be involved in specific cargo recruitment; e.g. there are two γCOP homologues in higher organisms, γ1 and γ2, as well as two ζCOP subunits, ζ1 and ζ2 [21], [24], [29]. With the exception of *εCOP (SEC28)*, yeast COPI components are strictly required for viability and inter-compartmental traffic [23], [30]–[31]; therefore, the formation of a functional COPI coat requires all the main subunits. Furthermore, the coatomer activity appears to be adapted to cell-type specific requirements. Secretion and Golgi functions are compromised in zebrafish mutants deficient for *α, β* and *β'COP*. In these mutants, the development of chordamesoderm cells proceeds abnormally [32].

Previously, we found that most COPI components are ubiquitously expressed during *Drosophila* development, as expected for proteins required for cell viability. They are expressed at higher levels in cells with secretory function, such as the salivary gland cells. During embryonic tracheal development, most coatomer subunits are expressed at elevated levels in tracheal cells [33]. These elevated tissue-specific expression levels might represent an adaptation to the increased needs for membrane recycling in secretory cells or cells undergoing morphogenesis and shape changes.

To find out more about the function of COPI-mediated membrane traffic during *Drosophila* development, we generated null mutations in the *γCOP* locus starting from a previously isolated *P*-element insertion into the *γCOP* locus [33]. In this study, we present the isolation of *γCOP* loss-of-function mutants and an analysis of the role of *γCOP* in the development of epithelial organs in the embryo. We show that *γCOP* null mutants die late in embryogenesis with a poorly differentiated cuticle, indicative of difficulties in secreting cuticle components. These mutants display defects in luminal secretion of several key proteins, which are required for the coordinated cell rearrangements and cell shape changes during tracheal tube morphogenesis. As a consequence, *γCOP* mutants show defects in cell rearrangements, in branch elongation, in tube dilation, as well as in tube fusion. We present genetic evidence that a specific subset of the tracheal defects in *γCOP* mutants is due to the reduced secretion of the Zona Pellucida protein Piopio because over-expression of this critical target rescues the tracheal branch elongation defects of *γCOP* mutants.

## Results

### Isolation of *γCOP* alleles

**Figure 1:**
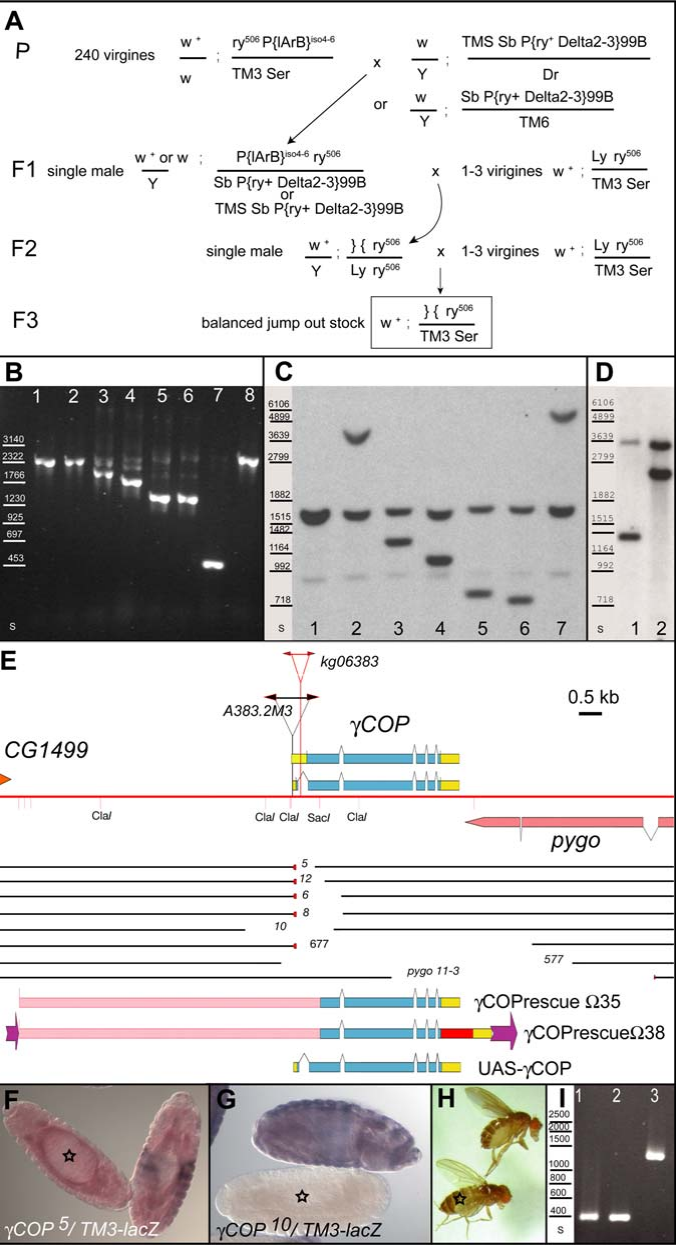
Structure of the *γCOP* locus and generation of *γCOP* mutations. (A) Strategy to generate *γCOP* jump out excision alleles from *γCOP^P{lArB}A383.2M3^*, which carries *ry^+^* as a selection marker. In the parental generation P, the *γCOP^P{lArB}A383.2M3^* line was crossed to any one of the *P*-element transposase lines, marked with *Sb*. In the F1 generation, single males undergoing *P*-element excision events were crossed to virgin *Ly ry^506^* females in order the chromosome, from which the *P*{lArB} has jumped out (marked }{ ), can be discriminated in the following generations from the homologous chromosome 3. The individual excision events were balanced in the F3 generation (TM3, *Ser*). (B) In lanes 1–8 PCR amplification products using primers gm4-cop6rev on genomic DNA of control and deletion lines was loaded; primers gm4-cop6rev amplify a fragment of 2305 bp from the *γCOP* locus of wild type or *ry^506^*. (1) *ry^506^*, (2) *γCOP^P{lArB}A383.2M3(iso6)^*/TM3 *Sb* (3) *γCOP^5^*/TM3 *Ser* (4) *γCOP^12^*/TM3 *Ser* (5) *γCOP^6^*/TM3 *Ser* (6) *γCOP^8^*/TM3 *Ser* (7) *γCOP^10^*/TM3 *Ser* (8) *ry^506^*. Standard molecular weights are indicated in lane s. (C, D) *Hind*III, *Eco*RI digested gDNA from control and deletion lines was probed with a DIG-labelled cop5-cop11rev fragment in (C) and with a 3prime1-3prime2rev fragment in (D) (see Materials and Methods); Standard molecular weights of DIG VII are indicated in lane s. The following genotypes were loaded and blotted in (C): *ry^506^* (1), *γCOP^P{lArB}A383.2M3^*/TM3 *Ser* (2), *γCOP^5^*/TM3 *Ser* (3), *γCOP^12^*/TM3 Ser (4), *γCOP^6^*/TM3 *Ser* (5), *γCOP^8^*/TM3 *Ser* (6), *γCOP^10^*/TM3 *Ser* (7). In (D) *γCOP^577^*/TM3 *Ser* (1), *γCOP^677^*/TM3 *Ser* (2). (E) Map of *γCOP* locus and the *γCOP^P{lArB}A383.2M3^* deletions. Chromosome 3R is indicated as a red line. *P*-element insertion of *γCOP^P{lArB}A383.2M3^* is indicated in black, *γCOP^kg06383^* insertion in red. Extent of the two alternative *γCOP* transcripts is shown, yellow boxes denote non-coding regions, blue boxes coding regions. cDNA LP01448 was used for cloning experiments (Materials and Methods). The 3'end of the neighboring gene *CG1499* is indicated above the red line. The 3'end of the neighboring gene *pygo* is indicated below the red line. The DNA present on the deletion chromosomes is indicated as black lines; the missing DNA in comparison to the original chromosome is a blank space; small red triangles indicate the parts of the *P*-element inverted repeat which stayed behind after P-element excision. In deletion *γCOP^10^* and *Df(3R)γCOP^577^* no P-element derived sequences have stayed behind. Constructs for transgenic flies are shown. The extent of the upstream genomic region present in rescue construct *Ω35* and *Ω38* is shown as a pink box; *Ω38* is tagged with mRFP (red box) and the *γCOP* 3'UTR (yellow box); in addition this construct is flanked by FRT sites (purple arrows); in *UASp-γCOP* the full length *γCOP* cDNA is present (see Materials and Methods). (F, G) Deletion alleles (balanced over TM3-*lacZ*) were analyzed for *γCOP* transcription with a DIG-labeled *γCOP* probe and a FITC-labeled βGal probe, to discriminate heterozygous (red and blue) from homozygous *γCOP* mutant embryos. Whereas deletion mutant *γCOP^5^* (marked with asterisk in (F)) still expresses *γCOP* (red staining), deletion mutant *γCOP^10^* (marked with asterisk in (G)) shows no *γCOP* transcripts. (H) Fly rescued with *γCOP* rescue construct (marked with asterisk) and heterozygous sibling fly (unmarked) are shown. (I) PCR amplification of primer gm4 and cop6rev on five rescued flies confirming the presence of the original deletion in the rescued viable adults; in the PCR reaction only the short amplicon of the deletion breakpoint is visible; Lane (s) shows standard sizes. The amplicon of *γCOP* mutant flies (*γCOP^10^ (1,2) γCOP^6^* (3)), rescued with insertion *Ω35-i7* (2) *or* insertion *Ω35*-*i8* (2, 3).

To investigate the function of *γCOP* during development, we determined the cellular and developmental defects of *γCOP* mutants. We previously identified a *P*-element insertion line within the *γCOP* locus, which maps to the haplo-insufficient region close to 100C (*γCOP^P{lArB}A383.2M3^*; [33]). *P{lArB}A383.2M3* was homozygous viable, weakly fertile and the flies were smaller than wild type. We considered the *P{lArB}A383.2M3* allele a weak hypomorphic allele of *γCOP,* as we expected a *γCOP* deletion to have more severe phenotypes ([30]; supporting information Text S1). We generated stronger *γCOP* mutants through remobilization of the *P*{lArB}A383.2M3 element, which is inserted within the 5'UTR of the *γCOP* transcription unit ([33]; Figure 1A, E; Supporting Information Text S1). By screening through a large number of embryonic lethal lines generated in the remobilization experiment using a PCR assay, we identified a few *γCOP* mutants harboring small deletions as well as others harboring larger deletions, which also remove parts of the neighboring gene *pygopous (pygo)* ([34]–[35]; Supporting Information Text S1). These seven lines were further investigated. Southern blot analyses confirmed the existence of physical deletions in all the different *γCOP* alleles (Figure 1B–D). Through sequence analysis, we determined the deletion breakpoints (Materials and Methods, Supporting Information Text S1). In the case of deletion *5*, *12*, *6*, *8* and *677*, a few base pairs of the 5'*P* inverted repeat sequence and in the case of deletion 6 also a few base pairs of unknown origin had stayed behind after the imprecise excision of the *P*{lArB}. In the case of *10* and *577,* the entire *P*{lArB} element, along with 5′ and 3′ adjacent sequences were excised (Figure 1E; Supporting Figure S1). We named these mutants *γCOP^5^, γCOP^12^, γCOP^6^, γCOP^8^, γCOP^10^, Df(3R)γCOP^577^* and *Df(3R)γCOP^677^* (for more details see Supporting Information Text S1). Whereas in *γCOP^12^, γCOP^5^* and *γCOP^6^* mRNA from the *γCOP* locus is still transcribed (Figure 1F; data not shown), no *γCOP* transcripts can be detected in homozygous embryos of the *γCOP^10^* allele (Figure 1G). Thus, we have not only identified deletions of the entire *γCOP* locus (*Df(3R)γCOP^577^* and *Df(3R)γCOP^677^*), but also a single mutant *γCOP* null allele (*γCOP^10^*), in addition to hypomorphic *γCOP* alleles (*γCOP^12^, γCOP^5^, γCOP^6^, γCOP^8^*; Supporting Information Text S1). The *γCOP* null allele (*γCOP^10^*) and the deletions removing the entire *γCOP* transcription unit (*Df(3R)γCOP^577^* and *Df(3R)γCOP^677^*), are embryonic lethal; complementation assays between the *γCOP* deletions (*6, 8* and *10*) and the *Df(3R)pygo^11-3^* [34] or the independent *γCOP^kg06383^* allele, which had become available in the meantime (Flybase), also confirmed that *γCOP* is indeed a gene essential for viability (data not shown). Thus, *γCOP* null mutations are recessive embryonic lethal, indicating that the *γCOP* locus does not represent the haplo-insufficient locus close to 100C on chromosome 3.

In the course of our deletion analysis, it became clear that there were additional mutations present on the *γCOP* deletion chromosomes, which could disturb a functional analysis of the *γCOP* mutants. Therefore, theses mutations were removed by meiotic recombination. Only cleaned chromosomes (e.g. *FRT82B sr^1^ e^s^ γCOP^10^* or *FRT82B e^s^ γCOP^10^*) were used in our further analyses (Materials and Methods; Supporting Information Text S1).

### *γCOP* zygotic mutants are embryonic lethal

**Figure 2:**
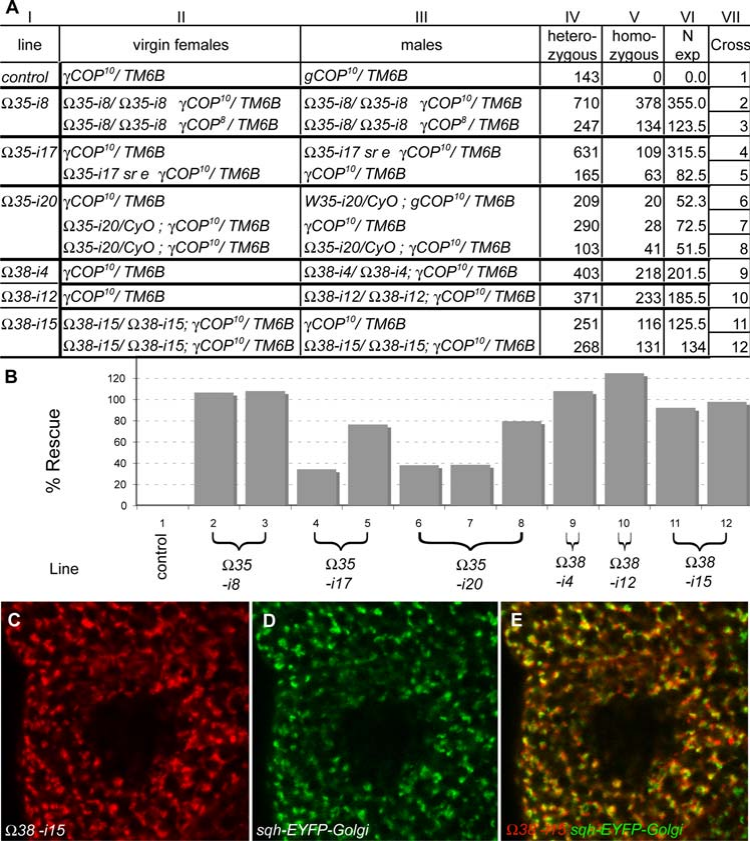
*γCOP* rescue constructs rescue lethality of *γCOP* mutants. Crossing different insertions of rescue construct *Ω35* or the *mRFP*-tagged rescue construct *Ω38* (*Ω35-i8, Ω35-i17, Ω35-i20, Ω38-4, Ω38-12, Ω38-15*) into the background of the *γCOP* null allele *γCOP^10^* (or other alleles) rescued the embryonic lethality of the *γCOP* mutants to different extents, depending on the line used. The different strength of the different insertion sites is likely due to position effect. (A) Columns: (I) rescue line used; (II) Parental genotype of the virgin; (III) Parental genotype of the male; (IV) Number of F1 flies, heterozygous and (V) number of F1 flies, homozygous for a given *γCOP* allele; (VI) Expected number of homozygous F1 flies if rescue was 100%; (B) Rescue activity for all crosses (column VII) is displayed as a bar chart. Line *Ω35-i17*, which was used in the tracheal rescue experiment (Figure 4, 5), shows already a significant rescue activity if paternally provided in one copy. The *mRFP*-tagged rescue construct *Ω38* rescues lethality of the *γCOP^10^* null allele even to 100%. (C, E) The *mRFP*-tagged rescue construct *Ω38* shows a punctate subcellular localization in living salivary gland cells, predominantly to the Golgi apparatus as visualized by an EYFP-Golgi marker (D, E Materials and Methods).

We first wanted to verify that the embryonic lethality and the associated phenotypes were indeed a consequence of the absence of *γCOP*. Therefore, we aimed to rescue the lethality of the different *γCOP* alleles using *γCOP* rescue constructs (*γCOPΩ35* and *γCOPΩ38)*. *γCOPΩ35* contains the entire *γCOP* coding sequence and also ∼5.8 kb of upstream sequence (Figure 1E). The first *γCOP* intron (which is only spliced out in the *γCOP-RA* mRNA) is present, whereas otherwise all introns are lacking in *γCOPΩ35* (Figure 1E; Materials and Methods). In our tests, several independent insertions of this *γCOP* rescue construct *Ω35* were found to rescue lethality of different *γCOP* alleles (e.g. *γCOP^8^* and *γCOP^10^*) to different extents (Figure 2). For example, the insertion *Ω35*-i8 on chromosome 2 fully rescued the lethality associated with *γCOP^10^*, when present in two copies (Figure 1H, Figure 2), while a single copy of insertion *Ω35*-i17 conferred a rescue activity of 76%. A similar rescue construct, which was tagged with mRFP at the C-terminus of *γCOP (Ω38),* was fully able to rescue the lethality of *γCOP* null mutants (Figure 1E, Figure 2). These experiments showed that *γCOP* fully accounts for the lethality associated with the deletion mutants and indicate that the associated phenotypes are due to the absence of *γCOP*. In addition, the mRFP-tagged rescue construct *(Ω38)* allowed us to inspect the sub-cellular localization of γCOP. Analyzing living salivary glands carrying both the mRFP-tagged construct *Ω38* and an EYFP-Golgi marker ([36], Materials and Methods) showed that γCOP predominately localizes to punctate structures, which correspond to the Golgi; such a subcellular localization of COPI components has also been observed in other organisms [22].

### *γCOP* is required for cuticle development

**Figure 3:**
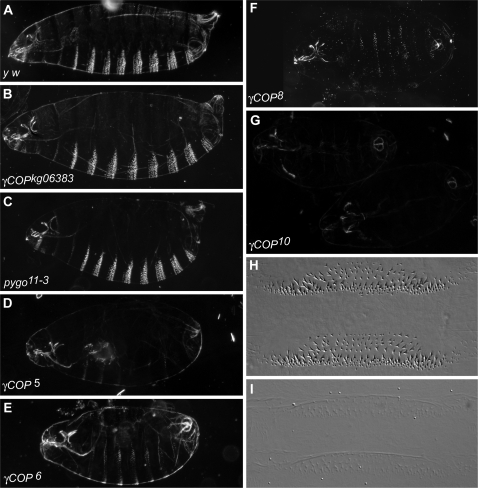
*γCOP* is required for cuticle development. Cuticle preparations of the following genotypes are shown: (A) *y w/y w*, (B) *γCOP^kg06383^*/*γCOP^kg06383^*, (C) *Df(3R)pygo^11-3^*/*Df(3R)pygo^11-3^*, (D) *P{neo^+^ FRT}82B γCOP^5^/P{neo^+^ FRT}82B γCOP^5^,* (E) *P{neo^+^ FRT}82B γCOP^6^*/*P{neo^+^ FRT}82B γCOP^6^*, (F) *P{neo^+^ FRT}82B γCOP^8^*/*P{neo^+^ FRT}82B γCOP^8^*, (G) *P{neo^+^ FRT}82B sr^1^ e^s^ γCOP^10^*/*P{neo^+^ FRT}82B sr^1^ e^s^ γCOP^10^*, (H–I) close-up of wt (H) and *P{neo^+^ FRT}82B sr^1^ e^s^ γCOP^10^*/*P{neo^+^ FRT}82B sr^1^ e^s^ γCOP^10^* (I) cuticle showing ventral side of the embryo (denticles).

To determine the lethal phase of *γCOP* mutants and the defects associated with a lack of zygotic *γCOP* function, we made cuticle preparations of the different *γCOP* alleles (Figure 3; Materials and Methods). All *γCOP* mutants die in late stages of embryogenesis. Presumably, the presence of maternal *γCOP* gene products [33] allows them to survive to such late stages. While embryonic patterning was rather normal in *γCOP* mutant embryos (see also below), they were smaller than wild type embryos and displayed weakly pigmented cuticles with poorly differentiated denticles (Figure 3); some of the mutants also displayed a partial dorsal open phenotype. The strongest phenotype was present in the embryos homozygous for the null allele *γCOP^10^*, which showed almost transparent cuticles and only weakly visible denticles (Figure 3G, I). The deletion alleles *γCOP^5^*, *γCOP^6^* and *γCOP^8^* are significantly stronger than the *γCOP^kg06383^* allele, but in comparison to the null allele, are hypomorphic for the cuticle phenotype, suggesting that these deletion alleles retain partial *γCOP* function (Figure 3B, D–F). It is conceivable that N-terminally truncated proteins are made from the RNAs of these hypomorphic deletion alleles (see Supporting Figure S1). Such truncated proteins might confer residual γCOP activity or a dominant negative activity, which would complicate the interpretation of the phenotypes of these alleles. Thus, they were not included in our subsequent investigation of tracheal development in *γCOP* mutants (see below). The phenotype of the homozygous *Df(3R)pygo^11-3^* allele, in which the C-terminal part of *γCOP* is missing, was also hypomorphic for the cuticle phenotype (Figure 1E; Figure 3C); it is conceivable that a 573 amino acid (aa) long γCOP protein is made in *Df(3R)pygo^11-3^* mutants (Supporting Figure S1). Notably, yeast mutants carrying a *γCOP* allele with a similar C-terminal deletion (579 aa) are not viable; expression of this truncated protein may also exert a dominant negative activity. Remarkably, a yeast strain expressing a slightly longer γCOP mutant protein (676 aa) shows temperature-sensitive lethality [37].

### *γCOP* is required for tracheal development

**Figure 4:**
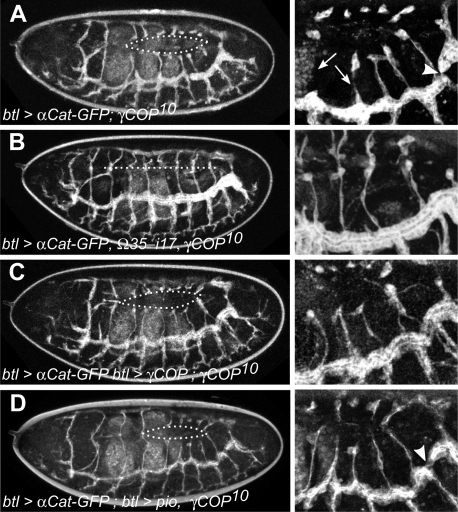
*γCOP* is required for tracheal tube morphogenesis. Live imaging of embryonic tracheal development in mutant and rescued embryos. Tracheal development was followed in live embryos using an *αCat-GFP* transgene specifically expressed in the tracheal system under the control of *btl*-*Gal4*. Pictures of late stage embryos were taken from movies (see supporting material) of the following genetic makeup: (A) *btl-Gal4 UAS-αCat-GFP; γCOP^10^*/*γCOP^10^*. (B) *btl-Gal4 UAS-αCat-GFP*; Ω35-17 *sr^1^ e^s^ γCOP^10^/* Ω35-17 *sr^1^ e^s^ γCOP^10^.* (C) *btl-Gal4 UAS-αCat-GFP/UASp*-*γCOP*; *γCOP^10^*/*γCOP^10^.* (D) *btl-Gal4 UAS-αCat-GFP*; *UAS-pio γCOP^10^*/*γCOP^10^.* Strikingly, the dorsal branches are frequently disrupted in *γCOP^10^* mutants (see arrows in A and Movie S1), similar to the phenotype observed in *pio* and *dp* mutants [8]. Dorsal trunk fusion defects are also observed in mutants (arrowhead in A). Both defects were rescued either by expressing a genomic *γCOP* rescue transgene (B), or by expressing *γCOP* specifically in the tracheal system using the UAS/Gal4 system (C). Tracheal-specific expression of Pio protein rescues the dorsal branch defects; however, dorsal trunk fusion defects are still visible (arrowhead in D).

**Figure 5:**
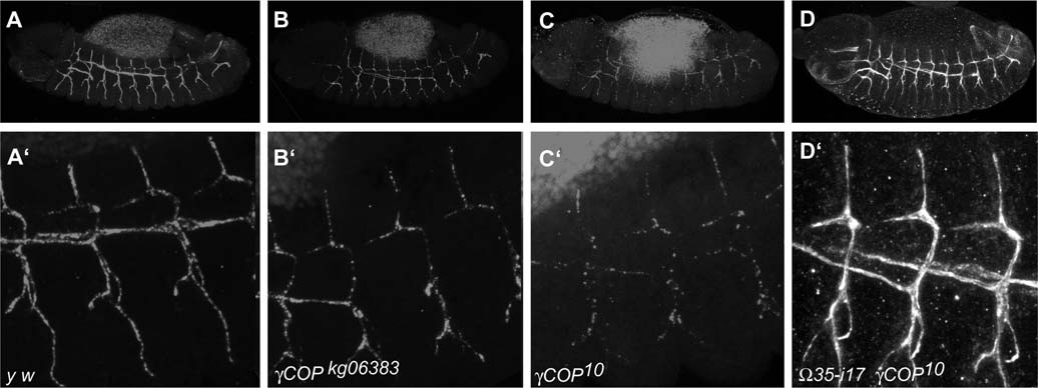
*γCOP* mutants are defective in Pio secretion. Pio levels are diminished in *γCOP^10^* mutants. Secretion of Pio into the lumen of the developing embryonic tracheae in control *y w* embryos (A, A' blow up). In the hypomorphic mutation *γCOP^kg06383^* there is only a mild effect on Pio secretion (B, B' blow up of homozygous *γCOP^kg06383^* mutant embryo of (B)). Only low levels of Pio staining are detectable in the lumen of *γCOP^10^* mutant embryos (C, C' blow up of homozygous *P{neo^+^ FRT}82B sr^1^ e^s^ γCOP^10^* mutant embryo of (C)); Secretion of Pio into the tracheal lumen is restored by *γCOP* expression from the Ω35 rescue construct (D', blow up of homozygous *Ω35-17 sr^1^ e^s^ γCOP^10^* embryo shown in (D)). In the hypomorphic mutation *γCOP^kg06383^* there is only a mild effect on Pio secretion (B, B' blow up of homozygous *γCOP^kg06383^* mutant embryo of (B)). Note that the gain in (C) was increased compared to (A, B, D) in order to show Pio signals (compare background yolk signals)).

Since we have previously observed that *γCOP* and most other coatomer subunits are expressed at elevated levels in tracheal cells, we analyzed tracheal development in *γCOP* mutants using live imaging and immunostaining (Figures 4 and 5). We observed a number of defects, shown in detail in Figure 4 and the corresponding supporting movies. While the branching pattern was similar or identical to wild type embryos, the dorsal branches were often disrupted and formed cyst-like structures rather than extended branches linked up to the dorsal trunk (DT; Figure 4A, Supporting Movie S1). These defects were rescued using the genomic rescue construct *Ω35* (Figure 4B; Supporting Movie S2) as well as upon trachea-specific expression of *γCOP* (Figure 4C, Supporting Movie S3, *UAS*-*γCOP*). The disruption of dorsal branches and the formation of cyst-like structures are reminiscent of the defects seen in *pio* and *dp* mutant embryos [8], suggesting that a lack of Pio and/or Dp might be the cause for these defects in *γCOP* mutants. To find out whether Pio was indeed reduced in *γCOP* mutants, we analyzed its expression using an anti-Pio antiserum (Figure 5; [8]). In wild type embryos, Pio protein accumulates in the tracheal lumen beginning at stage 13. Indeed, we found that the levels of Pio protein were slightly diminished in *γCOP^kg06383^* (Figure 5B, B'); reduction was more prominent in *γCOP^10^* homozygotes (Figure 5C, C'). These observations suggest that the reduced levels of Pio accumulation in the tracheal lumen in *γCOP* mutants cause the disruption of dorsal branches. To test this hypothesis, we over-expressed Pio specifically in the developing tracheal system in *γCOP* mutant embryos, and found that the cyst-like structures were not observed anymore; instead, all dorsal branches extended as they do in wild type embryos (Supporting Movie S4; Figure 4D; see also Figure 5D). Thus, reduced accumulation of Pio in the tracheal lumen in *γCOP* mutants causes a *pio*-like defect in dorsal branch formation.

**Figure 6:**
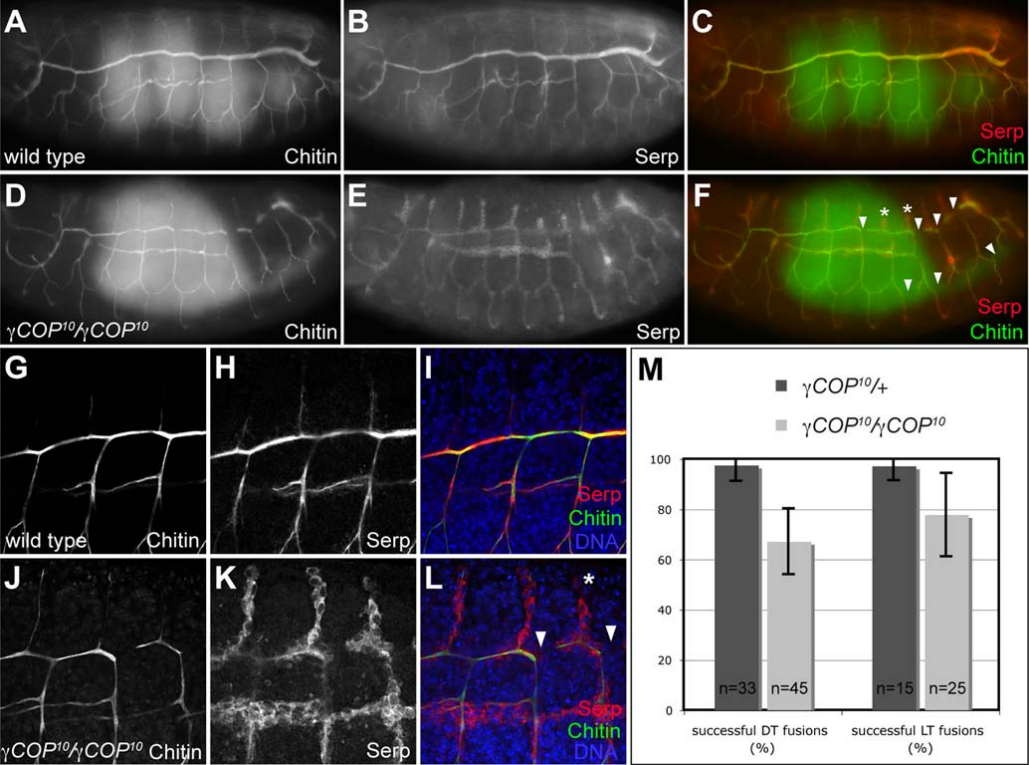
*γCOP* is required for apical protein secretion and tracheal lumen morphogenesis. (A–F): Chitin and Serp protein accumulate in the tracheal lumen of stage 16 wild type embryos (A–C). In contrast, Serp protein is retained in tracheal cells in *γCOP^10^* mutants, while luminal accumulation of chitin is not affected in the mutants (D–F). Note that the tracheal lumen marked by chitin staining in *γCOP^10^* embryos (D) is narrower than in wild type embryos (A). In addition, *γCOP^10^* embryos display interruptions in the DT lumen and in the LT (arrowheads in F) and stunted dorsal branches (asterisks in F) in posterior metameres. Also note that *γCOP^10^* embryos are developmentally delayed compared to wild type embryos, as indicated by gut morphology (green gut autofluorescence is visible in C, F). (G–L): Close-ups of wild type (G–I) and *γCOP^10^* (J–L) embryos. DT lumen fusion defects (arrowheads) and DB migration defects (asterisk) are indicated in (L). (M): Quantification of DT and LT fusion defects in *γCOP^10^* embryos (light grey bars) compared to heterozygous siblings (dark grey bars). 100% corresponds to nine successful fusion events in the ten tracheal metameres on one side of the embryo. Error bars indicate standard deviation. (A–F) are wide field fluorescent micrographs, (G–L) are single confocal sections taken at identical settings.

Closer inspection of the tracheal system in *γCOP* mutant embryos revealed that the dorsal trunk lumen was much narrower than in wild type embryos (see Figure 4A, and compare to Figure 4B). Since lumen expansion has been shown to rely on the secretion of a number of proteins into the luminal space [2], and since this additional tracheal phenotype was not rescued by Pio expression (and thus not due to the lack of Pio; see Figure 4D), we analyzed the expression of other luminal markers in *γCOP* mutants. In wild type embryos, the soluble secreted protein Serp accumulates in the tracheal lumen, where it associates with the luminal chitin cable (Figure 6A, B, G, H; [15]). While high levels of Serp are detectable in the tracheal lumen in wild type embryos, Serp protein is predominantly retained inside tracheal cells in *γCOP^10^* mutants (Figure 6E, K). Interestingly, Serp protein behaves differently from Pio protein; Pio is apparently secreted at lower levels, but is not detectable intracellularly in *γCOP* mutants (Figure 5C, C'). Chitin, which forms a cylindrical cable-like structure inside the lumen, is still found in the lumen in *γCOP* embryos, although at slightly reduced levels (Figure 6D, J). Taken together, *γCOP* is required for the accumulation of two secreted proteins, Pio and Serp, but not of the polysaccharide chitin, inside the tracheal lumen. To address the effects of reduced secretion at the morphological level, we analyzed tracheal morphology in more detail in *γCOP* mutants. In addition to the narrow lumen, *γCOP^10^* embryos displayed defects in DT lumen fusion, noticeable as interruptions in the luminal chitin cable in the DT (arrowheads in Figure 6F, L). These defects were variable in frequency (on average 3 DT lumen interruptions per side in *γCOP^10^* homozygotes (n = 45) compared to 0.2 interruptions in *γCOP^10^*/+ heterozygotes (n = 33); Figure 6M) and most frequently occurred in posterior segments. We also observed defects in lateral trunk (LT) fusion (Figure 6F, M). Together, these phenotypes are reminiscent of the tracheal fusion defects described for *Arl3/dead end* (*dnd*) mutants [12]–[13], suggesting that Arl3-mediated membrane remodeling during DT fusion is compromised in *γCOP* mutants. Thus, lack of *γCOP* causes defects in three distinct processes during tracheal development: dorsal branch elongation, lumen expansion and tube fusion.

## Discussion

In this study, we present the isolation of *Drosophila melanogaster γCOP* null mutations and the analysis of their effect on embryonic development. To obtain deletions within the *γCOP* locus, we remobilized a *P*-element insertion within the *γCOP* locus. Among the imprecise-excision deletions, we found a null mutant, which abrogates transcription from the *γCOP* locus, as well as two complete deletions of the *γCOP* CDS, which remove, in addition, parts of the distal neighboring gene *pygo.* Like in other organisms, the zygotic absence of *γCOP* is lethal [23], [30]. *γCOP* mutant embryos survive until late stages of embryogenesis, as was also shown recently in an independent study [39], likely due to the perdurance of maternal *γCOP* gene products, which are deposited in the egg during oogenesis [33]. These *γCOP* mutant embryos display defects in the formation of the embryonic cuticle; denticles are barely made. Judged by the severity of the cuticle phenotype, we classified the different mutations. As expected, the strongest defects were associated with the null allele *γCOP^10^*. Weaker mutant cuticle phenotypes were observed for the 5′ and 3′ deletions; this suggested to us that N-terminal or C-terminal truncated proteins may be made in these mutants, which retain residual *γCOP* activity. Studies in other organisms have shown that *γCOP* is an essential subunit of the COPI complex, which is involved in inter-compartmental traffic of small vesicles [21]–[22]. In the presence of truncated *γCOP* proteins, the heptameric COPI complex might still form and provide minimal, but not sufficient coatomer activity to the mutant cells. We expect that no COPI activity remains in cells harboring the *γCOP* null mutation alleles once they have run out of their maternal products, because the COPI complex most likely does not form in the absence of *γCOP* [37]. Furthermore, *γCOP* does not only interact with several of the other COPI subunits [37]–[38], it also represents one of the key interaction partners of coat assembly and disassembly regulators. It interacts with ARF1 and also p23/p24, which recruit coatomer to membranes [21]. *γCOP* also interacts with an ARF-GAP required for Golgi to ER retrograde trafficking vesicles [38]. Several different trafficking routes for the COPI complex have been proposed, which may be mediated through different isotypes of COPI subunits, including *γCOP* [29]. By mutating *Drosophila γCOP,* we expect to affect all the major coatomer-dependent traffic routes: *γCOP* is present as a single gene in *Drosophila melanogaster* and our N-terminal deletions remove the only alternative splice site known, which is not conserved in higher organisms [21], [33]. The cuticle phenotype of the *γCOP* mutants is similar, although stronger, than those described for mutants in other secretory pathway genes, *e.g*., *sec13* [40]. Sec13 is a component of the COPII complex involved in anterograde transport of small vesicles from the ER to the Golgi [40]–[41]. Therefore, a primary effect of removing *γCOP* functions from the embryo might be the inability to secrete proteins. Although coatomer has been predominantly implicated in retrograde transport of small vesicles, blocking retrograde transport should also affect anterograde transport. Membranes and the machinery required for vesicle formation and fusion are recycled back to the ER by means of the COPI-mediated vesicle transport from the ER-Golgi-Intermediate-Compartment (ERGIC), which is targeted by anterograde-moving, COPII complex-coated vesicles (see [41]–[42] and references therein). Indeed, in mutants of the yeast *γCOP* homologue *sec21,* ER to Golgi transport is affected [30]. Coatomer has also been implicated in transport of vesicles derived from Golgi cisternae [21]. Interestingly, Golgi functions are slowed down but not prevented in yeast mutants defective in COPI vesicle assembly [43]–[44]. Thus, COPI mutations could affect secretion in two ways: on the one hand, by slowing down the movement of cargo through the Golgi and on the other by blocking COPII-mediated transport due to the lack of recycling of proteins back to the ER, which are required for functions within the ER.

The idea that protein secretion is blocked in *γCOP* mutants is corroborated by our findings, as well as by recently published data [39], showing that several secreted proteins fail to accumulate to normal levels in the tracheal lumen of *γCOP* mutants. Serp protein levels in the tracheal lumen are severely reduced. Serp protein accumulates within the tracheal cells and the ZP-domain protein Pio is not present at normal levels in the tracheal lumen. Although small amounts of Pio protein are still found in the tracheal lumen of *γCOP* mutants, these residual amounts of protein appear to be insufficient to provide enough Pio function for proper epithelial tube morphogenesis. In the absence of *γCOP*, the dorsal tracheal branches are often disrupted, defects that are strikingly similar to those described for *pio* mutants [8]. Restoring function with a *γCOP* transgene in *γCOP* mutants rescues Pio secretion and DB migration, indicating that these defects are due to the lack of *γCOP* function. Interestingly, we were also able to restore DB integrity by over-expressing Pio specifically in tracheal cells in *γCOP* mutants. Thus, the requirement for *γCOP* in DB migration can be overcome by raising Pio protein levels, which presumably leads to increased levels of luminal Pio protein sufficient for normal cell intercalation. Importantly, this result suggests that Pio protein is the critical target whose reduced secretion in *γCOP* mutants is responsible for the specific DB defects observed in *γCOP* mutants. Thus, we were able attribute a specific subset of the defects in *γCOP* mutants to the failure in apical secretion of a distinct protein (Pio). This result was surprising, given that at least one additional protein (the ZP protein Dumpy) was previously shown to be required for DB cell intercalation along with Pio. However, Pio protein is required for luminal accumulation of Dp [8]. This suggests that the two ZP proteins are mutually dependent on each other for efficient transport through the secretory apparatus. Thus, raising the level of Pio protein in *γCOP* mutants may not only lead to increased secretion of Pio, but presumably also of Dumpy. Interestingly, a recent study using different, independently generated *γCOP* alleles [39] showed both by light and electron microscopy that *γCOP* is required for ER and Golgi structure, as well as for epithelial protein secretion. In addition, these authors showed that tracheal tube expansion is affected in *γCOP* mutants, while tracheal migration and fusion defects were not reported (see also below).

It was recently shown that targeted membrane remodeling in tracheal lumen fusion is dependent on the function of the exocyst complex [13]. Our finding that lumen fusion is also defective in *γCOP* null mutants raises the question whether the lumen fusion process is indirectly affected due to *γCOP's* general effect on luminal protein secretion, or whether the proper functioning of the exocyst complex and the lumen fusion process are affected more directly by the absence of COPI-mediated vesicle trafficking. We favor the latter scenario, because a general defect in COPII-dependent secretion in *sar1* mutants was shown to result in tracheal tube expansion defects similar to those observed in *γCOP* mutants, whereas tracheal lumen fusion was not reported to be affected in COPII component mutants [2]. Also, none of the secreted luminal proteins identified so far are required for lumen fusion. Thus, we argue that *γCOP* plays a more direct role in lumen fusion, maybe by affecting the function of the ARL3/Dnd G-protein [12]–[13]. *arl3* is expressed specifically in tracheal fusion cells, suggesting that it plays a dedicated role in membrane trafficking in the highly specialized tracheal tube fusion process. In contrast, *γCOP* and other components of the COPI complex are broadly expressed and are presumably generally required for COPI-dependent vesicle formation. Here, we show that the tracheal defects in *γCOP* mutants can be genetically dissected into (i) defects due to a general requirement for *γCOP* in all tracheal cells (luminal protein secretion, tube expansion) and (ii) defects due to a specific requirement in distinct cell types (dorsal branch cells, fusion cells).

## Materials and Methods

### *Drosophila* strains

*P{lArB}A383.2M3* is described in [33]. The *rucuca* chromosome (*roughoid^1^ (ru^1^)*, *hairy^1^ (h^1^)*, *thread^1^ (th^1^), scarlet^1^ (st^1^), curled^1^ (cu^1^), stripe^1^ (sr^1^) ebony^s^ (e^s^) claret^1^ (ca^1^)*) was isogenized before use in a standard meiotic recombination experiment; likewise the *P{neo^+^ FRT}82B* chromosome. The TMS *P{ry^+^ Δ2-3}99B* stock was a gift from Ulrich Schäfer. The *ry^506^ Sb P{ry^+^ Δ2-3}99B*/TM6 was a gift from Bill Engels. To balance the jump-out deletions a *Lyra (Ly) rosy (ry)*/TM3 Balancer was used. *UAS-pio* is described in [8]; *breathless (btl)-Gal4 UAS-αCatenin (αCat)-Green Fluorescent Protein (GFP)* is described in [45]. The Golgi maker *P{sqh-EYFP-Golgi}3* is described in [36] and was obtained from the Bloomington stock center. The following genotypes were used in Figure 1: *ry^506^, ry^506^ γCOP^P{lArB}A383.2M3^*/TM3 *Ser*, *ry^506^ γCOP^5^*/TM3 *Ser*, *ry^506^ γCOP^12^*/TM3 Ser, *ry^506^ γCOP^6^*/TM3 *Ser*, *ry^506^ γCOP^8^*/TM3 *Ser*, *ry^506^ γCOP^10^*/TM3 *Ser*, *ry^506^ γCOP^577^*/TM3 *Ser*, *ry^506^ γCOP^677^*/TM3 *Ser*. In Figure 2, the following lines were used: *y w*, TM2*/γCOP^kg06383^*, TM2/*Df(3R)pygo11-3*, TM2/*P{neo^+^ FRT}82B γCOP^5^*, TM2/*P{neo^+^ FRT}82B γCOP^6^*, TM2/*P{neo^+^ FRT}82B γCOP^8^*, TM2/*P{neo^+^ FRT}82B sr^1^ e^s^ γCOP^10^*, TM2/*γCOP^677^*.

### Single fly PCR

Genomic DNA from different candidate deletion lines was obtained using the method described by [46]. PCR analysis was carried out with different primer pairs amplifying smaller or bigger fragments spanning the original *P*{lArB} insertion site. For these “diagnostic” PCR reactions mainly the Red Taq DNA polymerase (Sigma) was used. The PCR strategy was similar to the one outlined below (determination of breakpoints), but adapted for the Red Taq polymerase.

### Determination of breakpoints

To amplify the deletion breakpoint of the *γCOP^6^* allele, the breakpoint sequence was amplified from genomic *γCOP^6^* DNA using either primers cop15 and cop6rev or cop14 and cop11rev together with the Advantage HF 2 PCR Kit (Clontech) and the following cycling conditions: 94°C for 1 min, then 35 cycles as follows: 94°C for 30 s, 60°C for 30 s, 68°C for 2 min. Cycling was ended with one round of incubation at 68°C for 5 min and then cooled to 4°C. Three PCR reactions were carried out in parallel and the products were gel-purified. Then they were pooled and directly sequenced using the nested primer cop14 for the first amplicon and primer cop6rev for the second. The breakpoint sequence of the other *γCOP* excision alleles was determined in a similar fashion; (sequencing primers cop14 for deletion *5*, *8*, *6*, *12*, *677*, primer cop15 for deletion *577*, primer cop6rev for deletion *10*); details are available upon request. Sequence data from this article have been deposited with the EBI/EMBL Data Libraries under accession numbers: AM398208 (*γCOP^5^*), AM398209 (*γCOP^12^*), AM398210 (*γCOP^6^*), AM398564 (*γCOP^8^*), AM503089 **(** *γCOP^10^*), AM398563 (*γCOP* ^677^), AM398565 (*γCOP* ^577^), EU447785 (*Df(3R)pygo ^11-3^*).

### Southern Analysis

Genomic DNA was isolated from different lines using a modification of the method described [47]; details are available upon request. It was digested using *Eco*RI and *Hind*III. Roughly 15 fly equivalents per slot were loaded on a 0.8% agarose gel and transferred onto a Hybond N^+^ Nylon membrane (Amersham) by the Alkali blotting procedure suggested by the manufacturer. Digoxigenin (DIG)-labeled probes were generated using the PCR DIG Probe Synthesis Kit (Roche); a fragment from cDNA LP01484 amplified using primers cop5 and cop11rev was used for the Southern Blot shown in Figure 1C. For the Southern Blot shown in Figure 1D, a fragment amplified with primers 3prime1 and 3prime2rev on genomic DNA, was used as a probe. The Southern blots were probed with the DIG-labeled fragments according to the instructions of the DIG Easy Hyb Granules manual (Roche) and developed according to the instructions of the CDP-Star manual (Roche). For a standard, the DIG-labeled DNA Molecular Weight Marker VII (Roche) was used.

### Oligonucleotides

R1 3'UTR (CGGAATTCTAAGACAGCGAGCCATGCA), 3'BamHI (CGCGGATCCCCTCCAATGGCCGCCGTTATCA), gm4 (GCGTGCGCCGACGTCTCAACTCTTG), cop2rev (CGATGATGACGTCCTCGGCAATGG), cop5 (GACCAGAGGATCTCTAAAGAGTCC), cop6rev (AAGCTCCTCAGAGGGAATGTCC), cop11rev (CACAGAGCGCAAATGGCCTGC), cop14 (ACAACATCGGAGCTATCGATAC), cop15 (CATCGCATTGATCAGCCTTCGC), 3prime1 (GCGGACTGTGCAGAGGAGGAGGAC), 3prime2rev (GACTACCGCCGCATATGAATCACG), 100 (GACTAGTGTCGACAAAACTCGGAGAGCCG), 101 (ACGCGTCGACCTCGAGCTTGCAATCATTAAAAT).

### Transgenes

Using the expand long Template PCR system (Roche), the genomic region of *γCOP* and a ∼5.8 kb region between *γCOP* and the proximal gene *CG1499* (ending at a *Sal*I site 3′ of the *CG1499* gene) was amplified from *ry^506^* flies according to the manufacturer's cycling conditions, using primer 100 for the 5′ end and primer 101 for the 3'end. This PCR product was subcloned into pCR 2.1 TOPO (Invitrogen). A *Sac*I fragment of the γCOP cDNA LP01448 was used to replace the sequences 3′ of the *γCOP*-internal *Sac*I site to the end. In this way, the entire rescue construct was framed by two *Not*I sites, which were used to retrieve this genomic-cDNA-hybrid rescue construct and to clone it into the *Not*I site of pPCaSpeR4 [48] resulting in p*P*{*w^+^* γCOP Ω35} (or *Ω35* for short notation). To create the monomeric Red Fluorescent Protein (mRFP)-tagged rescue construct p*P*{*w^+^ γCOP-mRFP Ω38*}, a *Bam*HI-*Eco*RI fragment containing *mRFP* [49] was subcloned into pCR 2.1 TOPO; An *Eco*RI site was introduced into the γCOP-3'UTR by PCR (primer R1 3'UTR and T7); this amplicon was cut with *Eco*RI and the *γCOP*-3'UTR was introduced into the *Eco*RI site of the *mRFP* clone. The stop codon of γCOP was changed to a *Bam*HI site by PCR: Using primer 3'BamHI and primer cop5, the 3'part of the γCOP coding region was amplified, cut with *Nar*I and *Bam*HI and cloned into the *Nar*I-*Bam*HI site of pKS-γCOP. To generate the γCOP-mRFP-γCOP 3'UTR-fusion, mRFP-γCOP 3'UTR was introduced as a *Bam*HI-*Xba*I fragment into the newly generated *Bam*HI site, which replaced the γCOP stop codon. The C-terminal γCOP-mRFP-γCOP 3'UTR fragment was introduced as a *Sac*I fragment (similar as in construct *Ω35*), into the γ*COP*-internal *Sac*I site of the genomic γ*COP* clone in pCR 2.1 TOPO. A 2 kb fragment containing the FRT-Flip-out casette [50], was introduced as *Asp*718 fragment into pPCaSpeR4-γCOP; subsequently one of the *Asp*718 sites and the γ*COP* insert were removed by *Xho*I restriction and religation, leaving the FRT-Flip-out cassette in pPCaSpeR4. Finally, the *mRFP-*tagged*-γCOP* transgene containing the genomic upstream region was recovered from pCR 2.1 TOPO as a *Not*I fragment and cloned into the internal *Not*I site of the FRT-cassette giving rise to rescue construct *Ω38* (see Figure 1E). For the pP{*w^+^* UASp-γCOP Ω31} construct, γ*COP* cDNA LP01448 (Research Genetics, [33]) was recovered as an *Eco*RV-*Ssp*I fragment und subcloned into the *Eco*RV site of pBluescript to add an *Asp*718 site at the 5′ end and a *Bam*HI site at the 3'end. Subsequently, the γ*COP* cDNA was cloned as an *Asp*718-*Bam*HI fragment into pPUASp [51]. All the *P*-element-based constructs were introduced into *y w* flies by *P*-element transformation [52].

### Whole mount *in situ* hybridization

The whole mount double *in situ* hybridization protocol and the probes are described [33].

### Cuticle preparation

Cuticle preparations of embryos were made essentially as described [53]. The *γCOP* mutations were balanced over a TM2 *Ubx* balancer chromosome to identify homozygous mutant embryos by the absence of the dominant *Ubx* phenotype, displayed by TM2 balancer chromosome carrying embryos.

### Antibody staining

Whole mount antibody staining was essentially carried out as described [33]. The rabbit anti-Pio antibody [8] was used at a dilution of 1∶200, the mouse monoclonal anti β−Galactosidase (anti-β−Gal; Promega) at 1∶500, rabbit anti-Serp and rabbit anti-Verm antibodies [15] were used at 1∶100. FITC- and Rhodamin-conjugated Chitin-binding probes (NEB Biolabs) were used at 1∶300.

### Quantification of tracheal fusion defects

Lumen fusion was analyzed in *γCOP^10^* homozygous embryos and heterozygous siblings by scoring the number of successful fusion events per ten tracheal metameres in stage 15 embryos stained for Chitin. Anti-β-Gal staining was used to genotype embryos.

### Live imaging

For live-imaging, embryos expressing *αCat-GFP* in the tracheal system under the control of the *breathless* promoter (*btl*-GAL4 *UAS−αCat-GFP*) were collected overnight, dechorionated in 4% bleach, and mounted in 400-5 mineral oil (Sigma Diagnostic, St Louis, MO, USA) between a glass coverslip and a gas-permeable plastic foil (bioFOLIE 25, InVitro System and Services, Göttingen, Germany). They were imaged either on a Leica TCS SP5 scanning confocal microscope or a Leica TCS SP scanning confocal microscope. Z stacks were collected every 2 min with optical sections at 1 µm intervals. Image processing was made with ImageJ (W Rasband; http://rsb.info.nih.gov/ij/) and house made ImageJ plugins. Z stacks were projected to get flat images. Salivary glands were dissected from third instar larvae in Grace's insect tissue culture medium (GIBCO) and were transferred to welled immunofluorescence slides (Polysciences), covered by a coverslip and analyzed by confocal microscopy.

## Supporting Information

Text S1 Identification of mutations and removal of background mutations.
(0.09 MB PDF)

Figure S1 Map of *γCOP* jump out deletions. Genomic sequence of the *γCOP* locus; position 1 has been chosen arbitrarily. *γCOP* transcripts LP01448 (for *γCOP*-RA) and the breakpoints of different *γCOP* jump out deletions are aligned. Exons are highlighted in bold. LP01448 starts at position 1086 and γCOP-RB starts at position 1067. Translation starting ATG sequences are displayed in capital letters and in red. The gt/ag consensus splice sites sequences are highlighted in red. It is notable that the first intron is spliced out only in the LP01448 transcript (highlighted in purple), therefore, the translation start site of the shorter transcript is present on the longer transcript (*γCOP*-RB) and seems to be ignored for the production of γCOP-PB. The protein sequence of both γCOP-PA and γCOP-PB are displayed below the genomic DNA sequence; the alternative N-terminus of γCOP-PB is highlighted in blue; Amino acid numbering is in black for PA and in blue for PB. The stop codons in all three frames in the 3'UTR are highlighted in red. In the deletion *γCOP^10^* positions 74 to 1974 are deleted (first and last present and deleted nucleotides are displayed as capital letters). In deletions *γCOP^5^*, *γCOP^12^*, *γCOP^6^* and *γCOP^8^* a short fragment of the P-element IR (magenta) and the transcription start site are still present. In *γCOP^6^* 28 bp of unknown origin are also present. The beginnings of the presumptive *γCOP* transcripts made in these deletions (starting with either the *γCOP*-RA or RB transcription start sequence) are shown in line with the genomic sequence at both breakpoints. The 5′ breakpoint for the deletions *γCOP^5^*, *γCOP^12^*, *γCOP^6^*, *γCOP^8^* is at position 1092, the 3′ breakpoint for the deletion *γCOP^5^* is at position 1568, the 3′ breakpoint for the deletion *γCOP^12^* is at position 1776, the 3′ breakpoint for the deletion *γCOP^6^* is at position 2139, the 3′ breakpoint for the deletion *γCOP^8^* is at position 2166. It is conceivable that a short peptide (MMK) is encoded on the IR sequence. However, it is also conceivable that a shortened γCOP protein is synthesized starting from the second AUG sequence present on these presumptive transcripts because in all deletions the second AUG is in frame with the coding frame and preceded by sequence motifs satisfying the requirements for a translation start site [54]–[55]. For all transcripts positions -1 to -4 (corresponding to the presumptive Shine-Dalgarno sequence) in respect to a potential translation starting AUG are underlined (red for wt *γCOP* transcripts, pink for the transcripts of mutant *γCOP* loci); nucleotides identical with the *Drosophila* consensus sequence as defined by Cavener [54] are displayed in capital letters. The deletion breakpoint of the *Df(3R)pygo^11-3^* allele is at position 3187 of the *γCOP* gene. Through the deletion, three new aa and a new stop codon are introduced; the *pygo^11-3^* locus gives potentially rise to a 570 aa *γCOP* protein ending with the sequence IMV (in total 573 aa).
(0.12 MB PDF)

Movie S1 Development of the tracheal system in a homozygous *γCOP^10^* mutant embryo. Anterior is to the left and dorsal to the top. The tracheal cells express *αCat-GFP*. The images were acquired at 2 min intervals. Several dorsal branches break. The dorsal trunk shows a fusion defect. Dorsal closure is incomplete.
(2.26 MB MOV)

Movie S2 Development of the tracheal system in a homozygous *γCOP^10^* mutant embryo carrying the genomic rescue construct *Ω35-i17*. Anterior is to the left and dorsal to the top. The tracheal cells express *αCat-GFP*. The images were acquired at 2 min intervals. Tracheal system development is normal. There is no dorsal closure defect.
(2.28 MB MOV)

Movie S3 Development of the tracheal system in a homozygous *γCOP^10^* mutant embryo expressing the *γCOP* cDNA LP01448 by means of the Gal4/UAS system in tracheal cells (UAS-*γCOP*). Anterior is to the left and dorsal to the top. The tracheal cells express *αCat-GFP*. The images were acquired at 2 min intervals. Tracheal system development is normal. However, dorsal closure is incomplete.
(2.29 MB MOV)

Movie S4 Development of the tracheal system in a homozygous *γCOP^10^* mutant embryo expressing *pio* by means of the Gal4/UAS system in tracheal cells (UAS-*pio*). Anterior is to the left and dorsal to the top. The tracheal cells express *αCat-GFP*. The images were acquired at 2 min intervals. The dorsal branches extend without breaking. The dorsal trunk still shows a fusion defect and does not expand normally. Dorsal closure is incomplete.
(2.28 MB MOV)
