# Supplementary material for: γCOP Is Required for Apical Protein Secretion and Epithelial Morphogenesis in Drosophila melanogaster
Source: PLoS One. 2008 Sep 19;3(9):e3241. doi: 10.1371/journal.pone.0003241 (PMC2532760; doi:10.1371/journal.pone.0003241)
Supplement: Text S1 — Identification of mutations and removal of background mutations. (0.09 MB PDF) [file pone.0003241.s001.pdf]

Supporting Information Text S1. Identification of mutations and removal of background mutations.

We generated three isogenic $\gamma COP^{P\{lArB\}A383.2M3}$ lines and found that these three lines were all homozygous viable (although in average only 31% of the expected homozygous flies were eclosing) and weakly fertile (egg-laying activity was lower than observed for wild type; data not shown). In addition, the flies were smaller and lacked the posterior cross-vein (data not shown). In our *P*-element remobilization experiment we started with all three different isogenic *P*{lArB}A383.2M3 lines and set up an initial 844 crosses, with single males, harboring both the *P*{lArB}A383.2M3 insertion and the *P*-element transposase (Figure 1A). Finally, the *P*-element remobilization experiment yielded 1108 stably balanced excision chromosomes, which had lost the ry^{+} marker and therefore likely the *P*{lArB}. Of these lines, 277 were lethal, the others were homozygous viable and crossveinless. Since *γCOP* had been found to be an essential gene in other organisms [30], we expected a *Drosophila γCOP* null mutant to be embryonic lethal; thus, novel *γCOP* alleles should be found among the lethal lines. By screening through a large number of these embryonic lethal lines using a PCR assay, we identified a few *γCOP* mutants harboring deletions (deletions *6, 8* and *10;* Figure 1B-C, E; Materials and Methods). In order to identify more and larger deletions, we were looking for suitable lines to be used in complementation assays: We used a jump-out deletion of the distal neighboring gene *pygo* [34], $Df(3R)pygo^{11\text{-}3}$, which also deletes parts of the *γCOP* gene (Figure 1E), a *pygo* allele, $pygo^{130}$, which specifically affects the *pygo* locus [34-35] and an independent *γCOP* allele, which had become available in the meantime, called *kg06383* (Flybase). By testing the remaining 144 embryonic lethal excision lines in complementation assays with $pygo^{130}$, $Df(3R)pygo^{11\text{-}3}$ and $\gamma COP^{kg06383}$ and our *PCR*-based assay, we identified additional *γCOP* and *γCOP pygo* double mutants (lines *5*, *12*, *577* and *677*

(Figure 1B-E)). Sequence analysis revealed the deletion breakpoints (Materials and Methods): In the case of *5*, *12*, *6*, *8* and *677*, a few base pairs of the 5'*P* inverted repeat sequence and in the case of deletion 6 also a few base pairs of unknown origin had stayed behind after the imprecise excision of the *P*{1ArB}. In γCOP^{5} 474 bp of the *γCOP* gene are deleted, in γCOP^{12} 682 bp, in γCOP^{6} 1045 bp, and in γCOP^{8} 1072 bp. In γCOP^{10}, sequences 5' of the known transcription start site, and sequences of the *γCOP* gene including both translation start sites (of γCOP-RA and RB) are deleted, in total 1900 bp (Figure 1E; Supporting Figure 1). Sequences 5' of the known transcription start site are also deleted in $Df(3R)\gamma COP^{577}$. In addition, $Df(3R)\gamma COP^{577}$ and $Df(3R)\gamma COP^{677}$ not only remove the entire *γCOP* transcription unit, but also parts of the 3' end of the distal neighboring gene *pygo* ([34-35]; Fig. 1E). In total 5405 bp are deleted in $Df(3R)\gamma COP^{677}$ and 6209 bp are deleted in $Df(3R)\gamma COP^{577}$ (Figure 1E).

We were also analyzing the homozygous viable lines, to find evidence that the *P*-element had precisely excised in these lines. Indeed by the single fly PCR method using primer pairs flanking the original *P*-element insertion (e.g. cop14 and cop2rev (Materials and Methods)), we revealed that in 51 of 93 (51/93) homozygous viable lines, the *P*-element had precisely excised. In only one line, which unfortunately was lost, a tiny deletion was detected. In another 10 lines, part of the *P*-element seemed to have remained in place as the PCR signal was bigger than in wild type; sequencing a few of these lines confirmed that only internal *P*-element deletions were present (data not shown). The remaining 31 lines were not further analyzed. This initial PCR analysis confirmed that in most homozygous viable lines the P-element had excised precisely or only small parts of it had stayed in place (and only the ry^{+} marker was lost). Sequencing two of these excision alleles confirmed that indeed the entire *P*-element was precisely removed (data not shown). However, these lines were still crossveinless (cv) and to our big surprise were

female sterile (fs). Crossing these excision lines to the lethal lines showed that most isolated lines were female sterile and crossveinless. This suggested to us that the crossveinless phenotype was due to a second mutation present in the original stock and that a third mutation was induced through the jump-out procedure. We considered this likely due to a second transposable element present in the background, which was not seen by our *in situ* hybridization experiment to polytene salivary gland chromosomes, using a *ry* probe [33]. In summary, not only *γCOP* deletion alleles but also *γCOP*-unassociated female sterile or potentially also lethal hits on chromosome 3 could be present in the lethal excision lines.

In complementation assays with the newly available independent *γCOP* allele *kg06383* (Flybase), we found that only 81 of the 144 of the lethal lines, as well as our PCR-identified *γCOP* deletions, did not complement *kg06383* and thus represent *γCOP* alleles. The others seemed to be lethal due to second hits present on chromosome 3, which were not associated with the *γCOP* locus. There is also no complementation observed between the $\gamma COP^{P\{lArB\}A383.2M3}$ allele and *kg06383* or the other newly identified *γCOP* deletions, indicating that the $\gamma COP^{P\{lArB\}A383.2M3}$ allele is indeed a hypomorphic *γCOP* allele (data not shown).

The presence of background mutations could severely disturb a functional analysis of *γCOP*. Therefore, the background mutations (*fs* and *cv*) were removed. We mapped them roughly by analyzing meiotic recombination events between the multiply marked *rucuca* chromosome (Materials and Methods, data not shown) and the *P{lArB}A383.2M3* and found the *cv* phenotype proximal to *e* and distal to *ry* (where two known *cv* mutations map *(cv-c* and *cv-d* (Flybase)) and the *fs* mutation on 3L. Then, we removed the background mutations by replacing all segments of 3L and all sequences proximal to e^s with an isogenic *rucuca* chromosome. This resulted in lines like e.g. ru^1 h^1 th^1 st^1 cu^1 sr^1 e^s γCOP^{10}. Furthermore, we removed most of the

rucuca makers again by recombination with either an isogenic FRT82B or another isogenic chromosome 3. In this way, lines like e.g. *FRT82B* sr^1 e^s γCOP^{10} or *FRT82B* e^s γCOP^{10} were obtained. Subsequently, these lines were tested in complementation assays to verify the absence of both the background *fs* and the *cv* mutation and used in our further analysis. Although we have replace almost the entire third chromosome, we can a priori not know whether potential lethal mutations have also been present or and if so, been removed, for they could principally be located distal to *ebony*. Therefore, rescue experiments were performed to prove that indeed no other lethal mutation than the one in *γCOP* was present on these chromosomes (Figure 2).
