# Supplementary material for: γCOP Is Required for Apical Protein Secretion and Epithelial Morphogenesis in Drosophila melanogaster
Source: PLoS One. 2008 Sep 19;3(9):e3241. doi: 10.1371/journal.pone.0003241 (PMC2532760; doi:10.1371/journal.pone.0003241)
Supplement: Figure S1 — Map of γCOP jump out deletions. Genomic sequence of the γCOP locus; position 1 has been chosen arbitrarily. γCOP transcripts LP01448 (for γCOP-RA) and the breakpoints of different γCOP jump out deletions are aligned. Exons are highlighted in bold. LP01448 starts at position 1086 and γCOP-RB starts at position 1067. Translation starting ATG sequences are displayed in capital letters and in red. The gt/ag consensus splice sites sequences are highlighted in red. It is notable that the first intron is spliced out only in the LP01448 transcript (highlighted in purple), therefore, the translation start site of the shorter transcript is present on the longer transcript (γCOP-RB) and seems to be ignored for the production of γCOP-PB. The protein sequence of both γCOP-PA and γCOP-PB are displayed below the genomic DNA sequence; the alternative N-terminus of γCOP-PB is highlighted in blue; Amino acid numbering is in black for PA and in blue for PB. The stop codons in all three frames in the 3'UTR are highlighted in red. In the deletion γCOP10 positions 74 to 1974 are deleted (first and last present and deleted nucleotides are displayed as capital letters). In deletions γCOP5, γCOP12, γCOP6 and γCOP8 a short fragment of the P-element IR (magenta) and the transcription start site are still present. In γCOP6 28 bp of unknown origin are also present. The beginnings of the presumptive γCOP transcripts made in these deletions (starting with either the γCOP-RA or RB transcription start sequence) are shown in line with the genomic sequence at both breakpoints. The 5′ breakpoint for the deletions γCOP5, γCOP12, γCOP6, γCOP8 is at position 1092, the 3′ breakpoint for the deletion γCOP5 is at position 1568, the 3′ breakpoint for the deletion γCOP12 is at position 1776, the 3′ breakpoint for the deletion γCOP6 is at position 2139, the 3′ breakpoint for the deletion γCOP8 is at position 2166. It is conceivable that a short peptide (MMK) is encoded on the IR sequence. However, it is also c [file pone.0003241.s002.pdf]

Supporting Figure S1. Map of *γCOP* deletion breakpoints

γCOP locus

```
                                                                                 1
                                                                                 |
                                                                                 ctt tgt agg tgt ttc cag ggg cca cag ctg
                                                         γCOP10
31                                       61               |                     91
|                                        |                |                     |
atg cct tga cag cta cta aat tct ccg ccc gtc tga cgc aca CGt tcc ttg tcc aat ttt ggg cac cag gcg gat ttt cct cct ggc cgg

121                                      151                                     181
|                                        |                                       |
cgc tta tcg cct ggc cat aaa ttc atg ctg tag gtg tct cga tct ttt cgg ttt ctt tct ttt ttg gct ttt tag cgt ttt ggc gga ggt

211                                      241                                     271
|                                        |                                       |
cct cgc ctc gtg gac acg caa tga agc tgt ttc ttc gtg cgc cgg aat ttc ccc gcg gca tgc aaa atg caa tta acg gca ctt gca gtg

301                                      331                                     361
|                                        |                                       |
cgc cgg aaa ttg aca att caa tat aat ggg ttc ctt gtt ttc gct aat tgg tgg tgc tta tgc acg cat aaa tta agt aaa cac aat acg

391                                      421                                     451
|                                        |                                       |
aag tcg tat tta att ggt caa aac aaa cta gag ctg aca aaa aag ctg gcg tcc att ttg tta aca gga tca aca ctc gat taa att agg

481                                      511                                     541
|                                        |                                       |
aaa agt aat cgg tac tta acg gtc acg aga tct atc gat cag aac ttc ccc tcc agc aaa tca taa atg tac taa tag cgt ata tta taa

571                                      601                                     631
|                                        |                                       |
ata taa acg aag tta tta tct aac taa aaa ttc atc gca ttg atc agc ctt cgc aat ctt cgt cac aat tcc ggc caa tgc cca ttt aaa

661                                      691                                     721
|                                        |                                       |
aaa cga atc aaa cta atg gta aaa aat tat gaa aat gta aat aaa att taa aac att att ata ttc ata caa ttt ctt agc aat ttc gtt

751                                      781                                     811
|                                        |                                       |
ata aag tta acg tgg agc aaa ttc aaa gct tag cta ttt ttc cgc cca ttc ata atc gat aat cta tcg ata aat aca atc gaa cca gta

841                                      871                                     901
|                                        |                                       |
agt gaa tct caa cta gtt cct ttt caa tcc agt gaa gaa aca aga aat ttt aag aac aaa aaa aac tac cag atc tcg gtg tcg agt taa

931                                      961                                     991
|                                        |                                       |
ttg aaa act gaa agt tat aat gtg cag aat cgg cga ata tat act gcc tac aaa acg cag aaa aaa ggt tgt ctt tta aag ata aga aca
```

```
                                                                                    γCOP8
                                                                                              gcug AguC AUG aug aaa ua ac auc ccc cag aua cgc..
                                                                                                         M   M   K   *

                                                                                    γCOP6
                                                                                              gcug AguC AUG aug aaa uaa caauaaagugaaauaaagugaaaagug...
                                                                                                         M   M   K   *

                                                                                    γCOP12
                                                                                              gcug AguC AUG aug aaa uaa cacggauaaCAcC AUG…
                                                                                                         M   M   K   *                M

                                                                                     γCOP5
                                                                      uga acc aac ugg gca ccg cug AguC AUG aug aaa uaa cauggcccgcgaggccaccgacugcuuuuu ugcg AUG.
                                                                                                         M   M   K   *                                           M
                                                                                                      |
                                                                                                      |
1021                                    1051                                    1081                  |
|                                       |                                       |                     |
aca tcg gag cta tcg ata ctt aac tgg agt ttt cac atc gat aac aga tga acc aac tgg gca ccg ctg agt gac gtg taa gcg cag cca

1111                                    1141                                    1171
|                                       |                                       |
gaa aac cat ttc aat tgt ttt acc cac aat tta acc gaa agt tgc aa gAcA ATG ggt tcg ttc cgc cgc gag aaa gat gat gag gag gac
                                                                    M   G   S   F   R   R   E   K   D   D   E   E   D
|                                       |                                       |
                                                                                4

1201                                    1231                                    1261
|                                       |                                       |
ggt aag tag caa gct agc acc tgg agc caa gtt gtc cgc cga cca gcg aag cca atc gaa gac aac cca gtt gct tga agg tgc tcc gct
|
14

1291                                    1321                                    1351
|                                       |                                       |
ggc aca gac aca cac tca cac acc cac taa ccc gac aaa gtc gag aaa aat cga ggt ttc cct aaa ttg act ga gAAC ATG aac tat ttt
                                                                                                         M   N   Y   F
1381                                    1411                                    1441
|                                       |                                       |
tcc ctt acc tcg cac aaa aaa cat cgc ggc cat ccg tca gca gga ccg agc aat gcg tac cag aac ctg gaa aaa acc tcg gtg ctt cag
S   L   T   S   H   K   K   H   R   G   H   P   S   A   G   P   S   N   A   Y   Q   N   L   E   K   T   S   V   L   Q
|                                       |                                       |
5                                       15                                   21/25

1471                                    1501                                    1531
|                                       |                                       |
gag acg cgc acc ttc aat gag acg ccg gtg aac cca agg aag tgc atc cac atc ctg acc aaa atc ctg tac ctg att aat cag ggt gag
E   T   R   T   F   N   E   T   P   V   N   P   R   K   C   I   H   I   L   T   K   I   L   Y   L   I   N   Q   G   E
|                                       |                                       |
31/35                                   41/45                                   51/55

ccgcugAguCaugaugaaauaaca ug gcc cgc gag gcc acc gac ugc uuu uuu gcg AUG - γCOP5
                        |
1561                    |               1591                                    1621
|                       |               |                                       |
cag ctt gtg gcc cgc gag gcc acc gac tgc ttt ttt Gcg ATG acg aag ctc ttc caa tct aag gac gtg gtg ctg cgt cgt atg gta tac
Q   L   V   A   R   E   A   T   D   C   F   F   A   M   T   K   L   F   Q   S   K   D   V   V   L   R   R   M   V   Y
|                                       |                                       |
61/65                                   71/75                                   81/85
```

```
1651                                    1681                                    1711
|                                       |                                       |
ctg ggc atc aag gag ctg agc tcc att gcc gag gac gtc atc atc gtg act agc tcg cta acg aag gat atg acc ggc aag gaa gac ctg
L   G   I   K   E   L   S   S   I   A   E   D   V   I   I   V   T   S   S   L   T   K   D   M   T   G   K   E   D   L
|                                       |                                       |
91/95                                   101/105                                 111/115

                       ccgcugagucaugaugaaauaa c acg Gau aaC AcC AUG - γCOP12
                                              |
1741                                    1771  |                                 1801
|                                       |     |                                 |
tac agg gcc gcc gca atc cgg gcc ttg tgc agc atc acg gat aaC AcC ATG ttg cag gcc gtg gag cgc tac atg aag cag tgc att gtt
Y   R   A   A   A   I   R   A   L   C   S   I   T   D   N   T   M   L   Q   A   V   E   R   Y   M   K   Q   C   I   V
|                                       |                                       |
121/125                                 131/135                                 141/145

1831                                    1861                                    1891
|                                       |                                       |
gac aaa aac gcg gca gtt tct tgt gct gca ttg gtc agt tct ttg aga ttg gcc aac act gcc ggc gac gtg gtc aag agg tgg gca aac
D   K   N   A   A   V   S   C   A   A   L   V   S   S   L   R   L   A   N   T   A   G   D   V   V   K   R   W   A   N
|                                       |                                       |
151/155                                 161/165                                 171/175

                                                                      γCOP10
1921                                    1951                            |        1981
|                                       |                               |        |
gag gcc cag gag gct ttg aac agc gac aac att atg gta cag tac cac gcg ttG Ggt ctg ctc tac cat atc cgc aag tcg gat cgg ctg
E   A   Q   E   A   L   N   S   D   N   I   M   V   Q   Y   H   A   L   G   L   L   Y   H   I   R   K   S   D   R   L
|                                       |                                       |
181/185                                 191/195                                 201/210

2011                                    2041                                    2071
|                                       |                                       |
gct gtc tcc aag ttg gtc aac aag ctg acc aga gga tct cta aag agt ccc tat gcc gtt tgc atg ctg gt a agt gaa gtc tga acc c
A   V   S   K   L   V   N   K   L   T   R   G   S   L   K   S   P   Y   A   V   C   M   L
|                                       |                                       |
221/225                                 231/235                                 241/245

 ccgcugAguCaugaugaaauaa caauaaagugaaauaaagugaaaaguga a aag uga cauc uga cua auu ucg uuu uac auc ccc cag aua cgc   γCOP6
                                                    |               ccgcugAguCAUGaugaaaua ac auc ccc cag aua cgc   γCOP8
2099                                    2129        |                       2159     |
|                                       |           |                       |        |
aaa ctt ttc tta ATG aa ATG gat ATG cac tta ttc cct tta aag tga catc tga cta att tcg ttt tac atc ccc cag ata cgc att gcc
                                                            *                                           I   R   I   A

2189                                    2219                                    2249
|                                       |                                       |
tgt aag ctg atc gag gag gag gac att ccc tct gag gag ctt tcc gat tca ccc ttg ttt acg ttc atc gag tcc tgt ctg cgc cac aag
C   K   L   I   E   E   E   D   I   P   S   E   E   L   S   D   S   P   L   F   T   F   I   E   S   C   L   R   H   K
|                                       |                                       |
248/252                                 258/262                                 268/272

2279                                    2309                                    2339
|                                       |                                       |
agC GAg ATG gtc atc tac gag gcg gcc cac gcc att gtc aac ctc aag aac acc aat ccg cga atg cta tcg ccg gcg ttc tcc atc ctc
S   E   M   V   I   Y   E   A   A   H   A   I   V   N   L   K   N   T   N   P   R   M   L   S   P   A   F   S   I   L
|       |                               |                                       |
278/282 |                               288/292                                 298/302
        M- γCOP6/γCOP8
```

```
2369                                    2399                                    2429
|                                       |                                       |
cag ctt ttc tgc agc tcg ccg aag gca acg ttg cgc ttt gcg gct gtg cgc acg ctt aac aag gtg gcg atg acg cac ccg gct gcg gtg
Q   L   F   C   S   S   P   K   A   T   L   R   F   A   A   V   R   T   L   N   K   V   A   M   T   H   P   A   A   V
2459                                    2489                                    2519
|                                       |                                       |
acc acc tgt aac ttg gac ctg gaa ggt ctc atc acg gac tcc aat cga tcg gtg gcc act ctg gcc atc act acg ctg cta aag acc ggc
T   T   C   N   L   D   L   E   G   L   I   T   D   S   N   R   S   V   A   T   L   A   I   T   T   L   L   K   T   G
2549                                    2579                                    2609
|                                       |                                       |
gct gaa tcc tcg gta gag cgt ctg atg aag cag atc tcc acg ttt gtg gcg gag att tcc gat gaa ttc aag gtg gtg gtc gtg cag gcc
A   E   S   S   V   E   R   L   M   K   Q   I   S   T   F   V   A   E   I   S   D   E   F   K   V   V   V   V   Q   A
2639                                    2669                                    2699
|                                       |                                       |
att tgc gct ctg tgc acc aaa tat ccg cgc aag cac aca gtg ctg atg aac ttc ctc agc ggt atg ctg cgc gag gag ggt ggc ctt gaa
I   C   A   L   C   T   K   Y   P   R   K   H   T   V   L   M   N   F   L   S   G   M   L   R   E   E   G   G   L   E
2729                                     2759                                   2789
|                                       |                                       |
tac aag acc tcc ata gtt gac acc atc atc acg atc atc gag gag aat gcg gac gcc aag gag tcc ggt ttg tcg cat ctg tgc gag ttc
Y   K   T   S   I   V   D   T   I   I   T   I   I   E   E   N   A   D   A   K   E   S   G   L   S   H   L   C   E   F
2819                                    2849                                    2879
|                                       |                                       |
atc gag gat tgt gaa cac gtt tca ctg gcg gtg agg att ctt cac ctg ctg ggc aag gag gga ccc ttt gcg gcc aca ccc tcc aag tat
I   E   D   C   E   H   V   S   L   A   V   R   I   L   H   L   L   G   K   E   G   P   F   A   A   T   P   S   K   Y
2909                                    2939                                    2969
|                                       |                                       |
ata cgc ttt atc tac aac cgc gtc atc ttg gag agc cct att gtg cga gca gcc gcg gtc acg gcc atg gcc cag ttt gga gca tct tgc
I   R   F   I   Y   N   R   V   I   L   E   S   P   I   V   R   A   A   A   V   T   A   M   A   Q   F   G   A   S   C
2999                                    3029                                    3059
|                                       |                                       |
cca gcc ttg tta agc aat atc ctc gtt ctg ctg ggt cgt tgc cag atg gac ccg gac gac gag gtg cgt gac cgt gcc acc tac tac ctt
P   A   L   L   S   N   I   L   V   L   L   G   R   C   Q   M   D   P   D   D   E   V   R   D   R   A   T   Y   Y   L
3089                                    3119                                    3149
|                                       |                                       |
agc att ctt aac tcg gaa agg cca gag ctg tac aag aac tac atc atc gaa agg gag aac tgt tct ctg gct ctg ctg gag aag tcg ctg
S   I   L   N   S   E   R   P   E   L   Y   K   N   Y   I   I   E   R   E   N   C   S   L   A   L   L   E   K   S   L
3179
|
GTC GAA CAT ATC ATG GTG TGA
V   E   H   I   M   V   *
|          |
|          | pygo 11-3
3179       |                            3209                                    3239
|          |                            |                                       |
gtc gaa cat ctg aat ggt gac gtg gac aca cgc ttc gat att tcc att gtg ccc aag gcg gcc ata gtt aag cca gtt att gcc aac gac
V   E   H   L   N   G   D   V   D   T   R   F   D   I   S   I   V   P   K   A   A   I   V   K   P   V   I   A   N   D
|                                       |                                       |
568/572                                 578/582                                 588/562

3269                                    3299                                    3329
|                                       |                                       |
gta atg cta gtt acc agc agt gct cca cgg ccg ccg aag atc acg cgc gag gag gag agt gct gca cgt ctg gca cag ctg ccg ggc atc
V   M   L   V   T   S   S   A   P   R   P   P   K   I   T   R   E   E   E   S   A   A   R   L   A   Q   L   P   G   I
3359                                    3389                                    3419
|                                       |                                       |
cag gtg ctg ggc ccc atc cat cgc agc aca gct ccc atc cag ctt act gaa agt gaa acg gag tac acg gtg cag tgc atc aaa cac ata
Q   V   L   G   P   I   H   R   S   T   A   P   I   Q   L   T   E   S   E   T   E   Y   T   V   Q   C   I   K   H   I
3449                                    3479                                    3509
|                                       |                                       |
ttc ggc cag cac gtg gtc ttc cag ttc gat tgc ttg aat acg ctg tcc gac caa atc ctt gaa aat gtg cgg gtg gaa ctc acg ctg ccg
F   G   Q   H   V   V   F   Q   F   D   C   L   N   T   L   S   D   Q   I   L   E   N   V   R   V   E   L   T   L   P
3539                                    3569                                    3599
|                                       |                                       |
gaa gga ttc aca acc agg gcg gtc att cca tgt ccc aag cta ccc tac aac gat ctg caa acc act ttt gtc atc gta gaa ttc ccg ccc
```

```
E   G   F   T   T   R   A   V   I   P   C   P   K   L   P   Y   N   D   L   Q   T   T   F   V   I   V   E   F   P   P
3629                                    3659                                    3689
|                                       |                                       |
gac gcc gcc aat tcc ata ggt aag ttt ata aat aac ata aat ttt gta tta aaa tca tac aac ttt ttt caa aac gaa cta ttt cct catt
D   A   A   N   S   I   A
3720                                    3750                                    3780
|                                       |                                       |
tcag cc acc ttt ggt gcc act tta cga ttt gtg gtc aag gac tgc gac ccc aac acc ggc gag ccg gag tca gag gag ggc tat gac gac
        T   F   G   A   T   L   R   F   V   V   K   D   C   D   P   N   T   G   E   P   E   S   E   E   G   Y   D   D
3810                                    3840                                    3870
|                                       |                                       |
gag tac atg cta gag gat ttg gag ctg acg gtt gcc gat cag ata cag aaa acc aga aag aac aat ttc caa gtg tcc tgg gat gcg gct
E   Y   M   L   E   D   L   E   L   T   V   A   D   Q   I   Q   K   T   R   K   N   N   F   Q   V   S   W   D   A   A
3900                                    3930                                    3960
|                                       |                                       |
gac agc gaa g gtgcc aca gaa aga taa ata ggg tct att caa tga tta gta ata ctc tat att tgcag aa tgg cta caa gcc gag gat acc
D   S   E   E                                                                                W   L   Q   A   E   D   T
3991                                    4021                                    4051
|                                       |                                       |
ttt gtg ctg tcg gca gtg acc acc ttg cag gat gcc gtc aac act ata gtc aag atc ctg ggc ttg ggc gct gca aac ctc tct gag aat
F   V   L   S   A   V   T   T   L   Q   D   A   V   N   T   I   V   K   I   L   G   L   G   A   A   N   L   S   E   N
4081                                    4111                                     4141
|                                       |                                        |
gtg ccc gag ggt acg cac ctg cat acg ttg ctc tgt tca g gt agg cat cct tcg ctt tta gtt ctg aga tca gta gta cag ttt ttc cc
V   P   E   G   T   H   L   H   T   L   L   C   S   G
4170                                    4209                                    4239
|                                       |                                       |
cct tta c ag ga acc ttc aga ggc ggc gcc gag att ctt gtg cgg gcc aag ctg gcg ctt tca gaa ggc gtc acg ctc aat ctg acg gtg
                T   F   R   G   G   A   E   I   L   V   R   A   K   L   A   L   S   E   G   V   T   L   N   L   T   V
4269                                    4299                                    4329
|                                       |                                       |
cgc agc acg gac cag gac gtg gcg gag ctg ata acg gcg gcc att gga taa gac agc gag cca tgc agg aac ctc ctt tgg ttc ctg cgt
R   S   T   D   Q   D   V   A   E   L   I   T   A   A   I   G   *
4359                                    4389                                    4419
|                                       |                                       |
gct gtg aac gta atc tga act ccc tac gaa act acg aaa cag aaa gtt acg cct aat caa aat ata att tgta cc tt taa gcga ctg tct
4449                                    4479                                    4509
|                                       |                                       |
ctg taa taa tct tag agc cct gtc cac aat ccg gca tgt gca aca ttg agt att gct act ggc cat ttc cga aaa gtt tgt gtg gcg atg
4539                                    4569                                    4599
|                                       |                                       |
cag cct tcg gtg tgc atc gca tat gtt tat tat ccc cat tta gcc tgt gca ttt agc tta agt atg gaa aca aaa tat taa aaa tac atg
4629                   4647             4659                                    4689
|                      |                |                                       |
tat atg caa tgc aac ccga cc tga agg cgt tta ttt ggc agg tgc ttt taa agt agg aca ttg aaa tga ttt taa tga ttg caa gct cgt
```
